# Whole Genome Sequence of Two Wild-Derived *Mus musculus domesticus* Inbred Strains, LEWES/EiJ and ZALENDE/EiJ, with Different Diploid Numbers

**DOI:** 10.1534/g3.116.034751

**Published:** 2016-10-07

**Authors:** Andrew P. Morgan, John P. Didion, Anthony G. Doran, James M. Holt, Leonard McMillan, Thomas M. Keane, Fernando Pardo-Manuel de Villena

**Affiliations:** * Department of Genetics, Lineberger Comprehensive Cancer Center, University of North Carolina, Chapel Hill, North Carolina 27599-7264; † Wellcome Trust Sanger Institute, Cambridge, CB10 1HH, United Kingdom; ‡ Department of Computer Science, University of North Carolina, Chapel Hill, North Carolina 27599-3175

**Keywords:** inbred mouse strains, wild-derived mouse strains, Robertsonian translocations, karyotype evolution

## Abstract

Wild-derived mouse inbred strains are becoming increasingly popular for complex traits analysis, evolutionary studies, and systems genetics. Here, we report the whole-genome sequencing of two wild-derived mouse inbred strains, LEWES/EiJ and ZALENDE/EiJ, of *Mus musculus domesticus* origin. These two inbred strains were selected based on their geographic origin, karyotype, and use in ongoing research. We generated 14× and 18× coverage sequence, respectively, and discovered over 1.1 million novel variants, most of which are private to one of these strains. This report expands the number of wild-derived inbred genomes in the *Mus* genus from six to eight. The sequence variation can be accessed via an online query tool; variant calls (VCF format) and alignments (BAM format) are available for download from a dedicated ftp site. Finally, the sequencing data have also been stored in a lossless, compressed, and indexed format using the multi-string Burrows-Wheeler transform. All data can be used without restriction.

*Mus musculus* (the house mouse) is among the most commonly used scientific model organisms (Didion and Pardo-Manuel de Villena 2013). “Classical” inbred strains and outbred mouse stocks used in research are primarily derived from a small founder population of *M. m. domesticus*, and therefore only sample a minor fraction of the genetic diversity present in the species as a whole (Yang *et al.* 2011; Keane *et al.* 2011). “Wild-derived” strains created by inbreeding of wild-caught mice have provided key phylogenetic context to mouse research. These relatively new strains are helping to revolutionize systems biology by increasing the resolution of genetic mapping studies, and by expanding the range of phenotypes and disease models available to researchers (Guénet and Bonhomme 2003; Phifer-Rixey and Nachman 2015). For example, studies of chromosomal abnormalities have greatly benefited from the availability of inbred strains derived from the chromosomal races of *M. m. domesticus*, which have fixed Robertsonian (Rb) translocations involving many different autosomes (Nachman and Searle 1995; Chmátal *et al.* 2014). Wild-derived strains have also enabled phylogenetic studies that improve our understanding of the relationships between classical strains and their wild ancestors (Ideraabdullah *et al.* 2004; Yang *et al.* 2011). Recently, a few wild-derived strains have been included among the selected few parental strains used to generate new panels of consomic lines (Gregorová *et al.* 2008; Takada *et al.* 2008) and popular genetics reference populations such as the Collaborative Cross (Collaborative Cross Consortium 2012) and the Diversity Outbred (Svenson *et al.* 2012; Chick *et al.* 2016).

However, the space of wild-derived inbred strains sequenced remains limited (CAST/EiJ, PWK/PhJ, SPRET/EiJ, MOLF/EiJ, MSM/Ms, and WSB/EiJ). Most taxa, including the *M. m. domesticus* subspecies, are currently represented by a single inbred strain—a limitation compounded by the fact that many wild-derived inbred strains carry intersubspecific introgressions (Yang *et al.* 2011).

Here, we report the whole-genome sequencing of two *M. m. domesticus* wild-derived inbred strains: LEWES/EiJ, which is derived from mice trapped in Lewes, DE, with the standard *M. musculus* diploid chromosome number of 40; and ZALENDE/EiJ, which is derived from mice trapped in the Poschiavinus Valley (Zalende, Switzerland), and has a 26-chromosome karyotype due to fixation of seven Rb translocations.

We sequenced LEWES/EiJ and ZALENDE/EiJ to enable several specific lines of inquiry. First, this effort triples the number of *M. m. domesticus* strains sequenced, and thus provides a clearer picture of intrasubspecific variation. LEWES/EiJ and ZALENDE/EiJ were among the top 10 inbred strains recommended for resequencing based on their potential to increase the catalog of known mouse variants (Kirby *et al.* 2010).

In addition, Rb translocations have been implicated in the evolution of the mammalian karyotype, and are subject to meiotic drive during female meiosis in both mouse and humans (Pardo-Manuel de Villena and Sapienza 2001b). Our laboratory is interested in the chromosomal races of *M. m. domesticus* (Gropp and Winking 1981; Qumsiyeh 1994; Piálek *et al.* 2005; Garagna *et al.* 2014) as a model of nonrandom chromosome segregation, karyotype evolution, and the mechanisms underlying the relatively high rates of aneuploidy and trisomy in humans (Pardo-Manuel de Villena and Sapienza 2001a). We were particularly interested in whether fixation of multiple Rb translocations (*e.g.*, in ZALENDE/EiJ) is associated with losses or gains on the centromeric ends of the chromosomes involved.

LEWES/EiJ was selected because it has been used extensively in studies of male sterility in *M. m. domesticus* × *M. m. musculus* hybrids (Good *et al.* 2008). Knowledge of the specific alleles carried by LEWES/EiJ at key hybrid sterility loci will guide interpretation of those studies.

Finally, we recently discovered a large copy number variant on Chromosome 2, *R2d2*, for which the high copy number allele is associated with distorted transmission ratios in heterozygous female carriers (Didion *et al.* 2015). Importantly, we found that *R2d2* has driven selective sweeps in the absence of fitness gain (“selfish sweeps”) in multiple independent mouse populations (Didion *et al.* 2016). *R2d2* was first identified in WSB/EiJ, a *M. m. domesticus* strain derived from mice trapped in Centreville, MD. SNP genotyping data (Yang *et al.* 2011; Didion *et al.* 2012; Morgan *et al.* 2016a) indicated that ZALENDE/EiJ harbors the high-copy (*i.e.*, distortion-associated) *R2d2* allele, while LEWES/EiJ—derived from mice trapped only ∼100 km away from Centreville—has the low-copy (*i.e.*, wild-type) *R2d2* allele. We hypothesized that comparison of the WSB/EiJ genome, which was sequenced previously (Keane *et al.* 2011), to the LEWES/EiJ and ZALENDE/EiJ genomes would help to fine-map *R2d2*, and characterize its evolutionary history (Morgan *et al.* 2016b), and could be of further use in understanding the relationship between copy number and transmission distortion at this locus.

Here, we describe the basic characteristics of these two genomes, make them available for public use, and discuss how this resource may benefit the community.

## Materials and Methods

### Mouse strains

#### LEWES/EiJ (https://www.jax.org/strain/002798):

Derived from wild mice trapped in Lewes, DE. Mice were sent from Michael Potter (National Cancer Institute) to Eva M. Eicher at The Jackson Laboratory in 1996.

#### ZALENDE/EiJ (https://www.jax.org/strain/001392):

Derived from mice trapped in the Poschiavinus Valley (Zalende, Switzerland) by Richard D. Sage. Mice from Sage’s colony were transferred to Michael Potter in 1981. A single pair of mice was sent from Michael Potter to Eva M. Eicher at The Jackson Laboratory in 1982. ZALENDE/EiJ is homozygous for seven Rb translocations (1.3, 4.6, 5.15, 11.13, 8.12, 9.14, and 16.17).

### Sequencing

High molecular weight DNA from one ZALENDE/EiJ male was obtained from The Jackson Laboratory. High molecular weight DNA from a LEWES/EiJ female was prepared from tissues samples from a colony maintained in the Pardo-Manuel de Villena laboratory in the past (Bell *et al.* 2006)

Libraries were prepared and sequenced at the University of North Carolina High Throughput Sequencing Facility. Genomic DNAs were sheared by ultrasonication and the resulting fragments were size-selected to target size 350 bp using a PippinPrep system. Samples were barcoded (LEWES/EiJ, two barcodes; ZALENDE/EiJ, four barcodes), pooled, and sequenced across multiple lanes and multiple flowcells on an Illumina HiSeq 2000 instrument. After a small pilot run with single-end 50-bp reads, and a mixture of single- and paired-end 100-bp reads were generated for each sample. Base calling and demultiplexing were performed using the Casava 1.8 pipeline. We obtained 498,668,400 reads for LEWES/EiJ and 573,861,165 reads for ZALENDE/EiJ.

### Data processing

Integrity of raw sequencing reads was confirmed using FastQC (Andrews 2010). Reads were aligned to the mm10 reference genome using bwa mem v0.7.5a–r406 (Li 2013). Coverage and quality summaries were computed using the Picard suite (http://broadinstitute.github.org/picard).

SNV and short indel variants were called using the Sanger Mouse Genomes Project pipeline, the current version of which is described in detail elsewhere (Doran *et al.* 2016). Briefly, samtools mpileup v1.1 and bcftools call v1.1 were used to generate an initial call set for the nuclear genome and mitochondrial genome. Candidate variants were filtered on the basis of read depth (> 5, < 100 for nuclear genome; > 350 for mitochondrial genome), mapping quality (> 20), number of reads supporting the alternate allele (> 5), proximity to an indel (> 2 bp; SNVs only) and homozygosity. Variants were declared private to a strain if the alternate allele was absent from all 30 other strains in the Mouse Genomes Project catalog. Analyses presented in this manuscript were performed only on variants passing all filters.

### Burrows-Wheeler transforms

The multi-string Burrows-Wheeler transform (BWT) for each whole genome dataset was individually constructed in-memory using the ropebwt2 program (Li 2014). After construction, each BWT was encoded using the run-length encoding format of the msbwt program (Holt and McMillan 2014) for disk storage. The BWTs for LEWES/EiJ and ZALENDE/EiJ are 9.6 and 10.1 GB in size, respectively. Instructions for building BWTs are publicly available at https://github.com/holtjma/msbwt/wiki/Converting-to-msbwt’s-RLE-format.

### Data availability

Raw reads have been deposited in the European Nucleotide Archive (accession #PRJEB15190). All processed data are available from the Sanger Mouse Genomes Project (http://www.sanger.ac.uk/science/data/mouse-genomes-project). Aligned reads (in BAM format): ftp://ftp-mouse.sanger.ac.uk/current_bams; web interface for querying variants: http://www.sanger.ac.uk/sanger/Mouse_SnpViewer/rel-1505; bulk download of variants (VCF format): ftp://ftp-mouse.sanger.ac.uk/current_snps). BWTs are available for download at http://csbio.unc.edu/WildDerived.

## Results and Discussion

**Figure 1:**
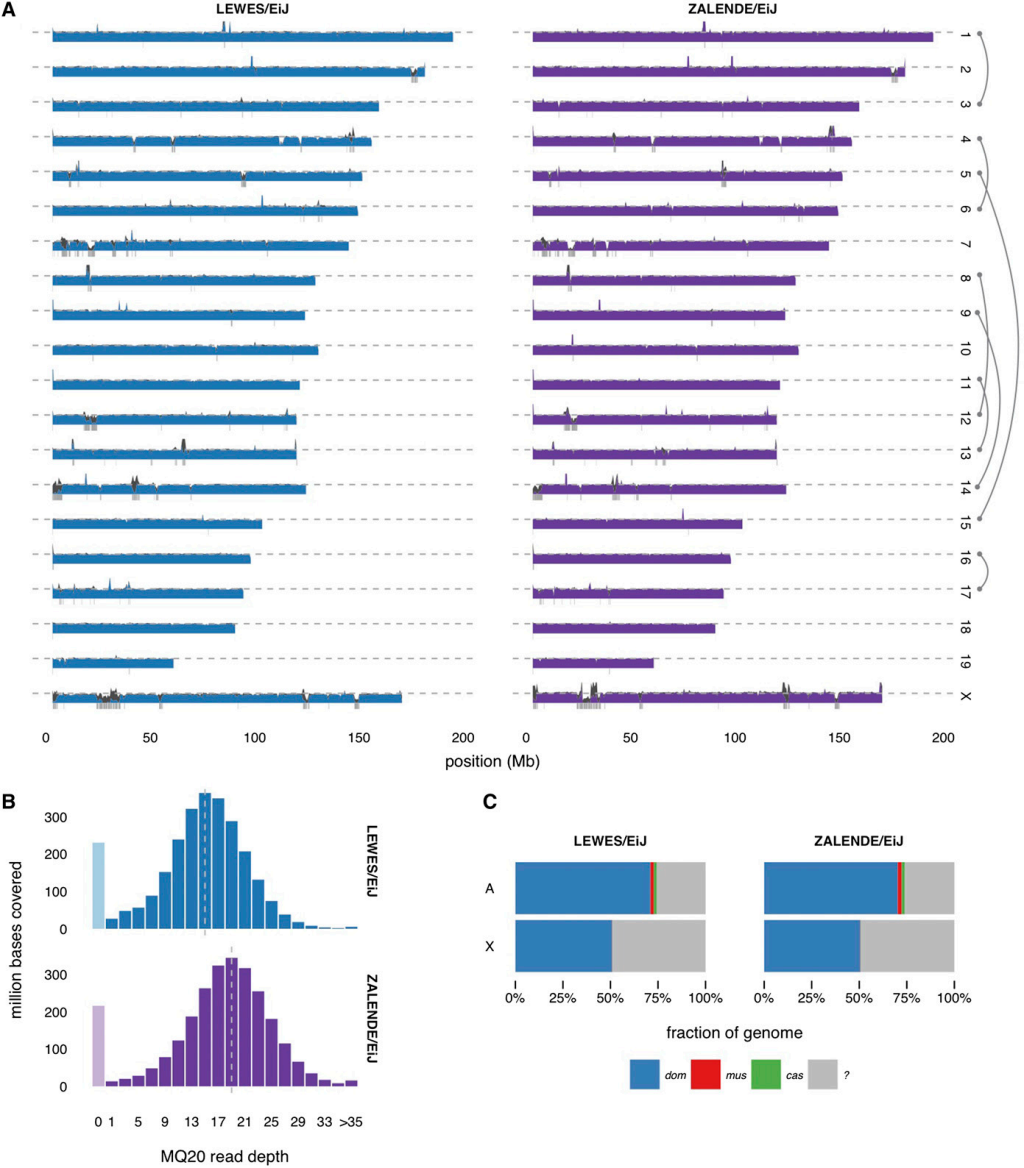
Sequencing coverage. (A) Profiles of normalized read depth across autosomes and X chromosome. Blue and purple regions show coverage by reads with mapping quality (MQ) > 20; dark gray regions show coverage by reads with MQ < 20. Gray dashed line indicates expected haploid depth. Boxes below axis show positions of segmental duplications > 100 kb in the mm10 reference genome. Note that the results for ZALENDE/EiJ are projections onto the reference genome, since this strains has only 13 chromosomes; chromosome pairs involved in Robertsonian fusions are indicated at right. (B) Histograms of coverage by reads with MQ > 20 across the genome. Gray dashed line indicates median coverage in each sample. (C) Estimated ancestry proportions (dom, *M. m. domesticus*; mus, *M. m. musculus*; cas, *M. m. castaneus*; ?, masked) on autosomes and X chromosome.

We sequenced one female LEWES/EiJ individual to median 14× coverage (overall alignment rate 99.5%), and one male ZALENDE/EiJ individual to median 18× coverage (alignment rate 99.4%). Genetic sex was confirmed by comparing relative read depth on the X chromosome to the autosomes. Coverage profiles across the nuclear genome are shown in Figure 1A; corresponding histograms appear in Figure 1B. After filtering reads with ambiguous alignments or poor base-call quality, coverage of at least 10× was achieved over 77.2 and 84.7% of the genome in each sample, respectively. This represents the fraction of the genome accessible for identification of sequence variants. The remaining fraction of the genome lies almost entirely in repetitive elements and clusters of polymorphic segmental duplications (*e.g.*, the proximal regions of chromosomes 7 and 14) where unambiguous alignment of short reads is not possible.

Sequence variants (SNVs and short indels < 10 bp in size) were ascertained using the Sanger Mouse Genomes Project pipeline (Doran *et al.* 2016). We identified 1,119,052 SNVs that have not been previously reported in any other mouse inbred strain (Keane *et al.* 2011; Doran *et al.* 2016). Of those variants, 102,561 are shared exclusively by the two strains, while 403,770 and 612,721 SNVs are private to LEWES/EiJ and ZALENDE/EiJ, respectively (Table 1), with a transition:transversion ratio of 2.14. Comparison of these totals with the number of unique variants discovered in other inbred strains reveals that sequencing of wild-derived inbred strains of *M. m. domesticus* origin identifies at least one order of magnitude more variants that sequencing classical laboratory strains (Table 2) (Keane *et al.* 2011; Doran *et al.* 2016).

**Table 1:** Variant-calling statistics

	LEWES/EiJ		ZALENDE/EiJ		Shared	
Private SNVs	403,770		612,721		102,561	
Coding	3619	0.90%	5525	0.90%	672	0.66%
Damaging	39	0.01%	67	0.01%	3	0.00%
Private indels	92,082		157,366		19,684	
Coding	51	0.06%	86	0.05%	9	0.05%
Damaging	49	0.05%	77	0.05%	7	0.04%

**Table 2:** Deletions of protein-coding genes

Strain	Ensembl Gene ID	Gene Symbol	Chromosome
LEWES/EiJ	ENSMUSG00000070868	*Skint3*	4
	ENSMUSG00000055960	*Skint4*	4
ZALENDE/EiJ	ENSMUSG00000073609	*D2hgdh*	1
	ENSMUSG00000094651	*Gal3st2*	1
	ENSMUSG00000093805	*Gm9994*	1
	ENSMUSG00000089951	*Gm14435*	2
	ENSMUSG00000070868	*Skint3*	4
	ENSMUSG00000055960	*Skint4*	4
	ENSMUSG00000049972	*Skint9*	4
	ENSMUSG00000055594	*5530400C23Rik*	6
	ENSMUSG00000067599	*Klra7*	6
	ENSMUSG00000091620	*Vmn2r23*	6
	ENSMUSG00000094298	*Gm6164*	7
	ENSMUSG00000094981	*Gm8653*	7
	ENSMUSG00000093941	*Vmn1r131*	7
	ENSMUSG00000091195	*Gm17332*	11
	ENSMUSG00000091275	*Gm3248*	14
	ENSMUSG00000096345	*Esp16*	17
	ENSMUSG00000079342	*Lipo1*	19
	ENSMUSG00000079387	*Luzp4*	X

Of the 1.1 M new variants, 0.88% fall within coding sequences and three (shared), 39 (private to LEWES/EiJ), and 67 (private to ZALENDE/EiJ) are predicted to disrupt gene function. We observe a similar picture for small indels (Table 1). As expected, the number of small indels that fall with coding exons is smaller but the proportion of predicted damaging mutations is higher.

We also identified large deletions that are predicted to encompass multiple exons of at least 20 genes (Table 2). These deletions can be ascribed to 11 events, and represent natural knockouts that are compatible with life in a laboratory setting. Most of them affect members of large and highly polymorphic gene families. Those that affect single-copy genes of well-defined function such as *D2hgdh* and *Gal3st2*, are also present in other sequenced strains (Keane *et al.* 2011, Doran *et al.* 2016). Interestingly, both the number of deletion events, and genes deleted, appears to be higher in ZALEDE/EiJ than in LEWES/EiJ. We speculate that differences between the two strains in both their phylogenetic proximity to the C57BL/6J reference sequence, and the effective population sizes of their wild progenitors, may explain these differences.

**Figure 2:**
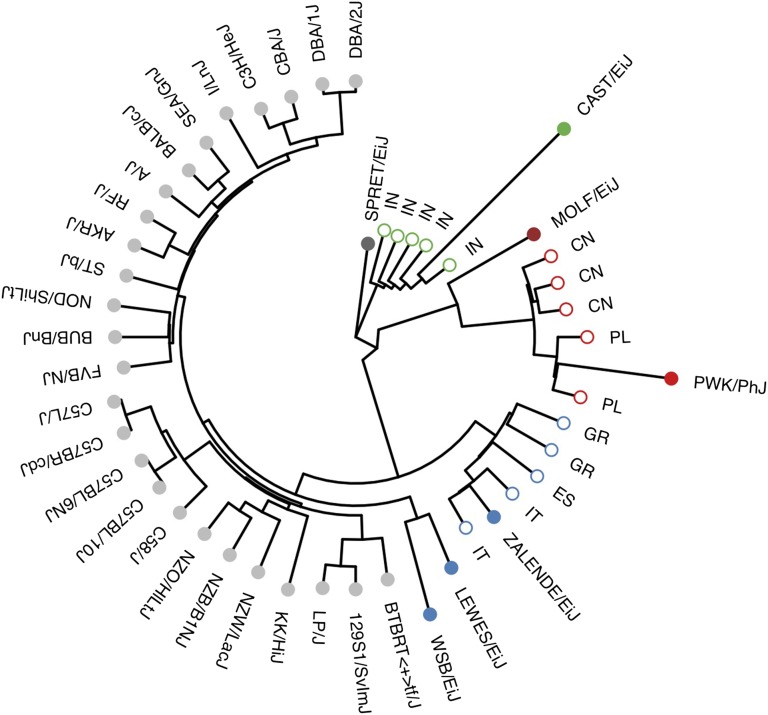
Phylogenetic tree of Mouse Genomes Project strains. Tree was constructed from genotypes at 30,000 ancestry-informative SNPs from the Mouse Diversity Array identified in Yang *et al.* (2011). Filled dots, inbred strains sequenced by Sanger MGP; open dots, wild-caught mice from Yang *et al.* (2011), identified by two-letter country code. Samples are colored according to subspecies of origin: blue, *M. m. domesticus*; red, *M. m. musculus*; maroon, *M. m. molossinus* (MOLF/EiJ); green, *M. m. castaneus*. The tree was rooted using SPRET/EiJ (*M. spretus*) as the outgroup.

Both LEWES/EiJ and ZALENDE/EiJ were reported to have essentially pure *M. m. domesticus* ancestry based on genotypes from the 600 K SNP Mouse Diversity Array (Yang *et al.* 2011; Figure 2). We sought to confirm this result using dense genotypes from whole-genome sequencing. We used three wild-derived strains already sequenced by the Mouse Genomes Project—WSB/EiJ (*M. m. domesticus*), PWK/PhJ (*M. m. musculus*) and CAST/EiJ (*M. m. castaneus*)—as representatives for their subspecies, and SPRET/EiJ (*Mus spretus*) as an outgroup. We classified alleles as ancestral or derived based on the pattern of sharing with the outgroup. After masking the 19% of the genome with known intersubspecific introgression or contamination in these strains (Yang *et al.* 2011), we calculated (in 25-kb windows) the proportion of derived alleles shared between LEWES/EiJ and exactly one of WSB/EiJ, PWK/PhJ, or CAST/EiJ, and repeated this analysis with ZALENDE/EiJ. Both strains share a majority of derived alleles with WSB/EiJ (*M. m. domesticus*) over 92 and 94% of the genome, respectively (Figure 1C). The remaining 8 and 6% of windows represent either introgression from *M. m. musculus* or *M. m. castaneus* in the two wild-derived inbred strains sequenced here; small *M. m. domesticus* introgressions into PWK/PhJ and CAST/EiJ (Wang *et al.* 2012); regions of incomplete sorting of ancestral polymorphism among the three subspecies (Keane *et al.* 2011); homoplasy (recurrent mutation); or some combination of the these.

Finally, we investigated whether the Robertsonian translocations in ZALENDE/EiJ are associated with large deletions or duplications near the centromeres of the affected chromosomes (1, 3, 4, 5, 6, 8, 9, 11, 12, 13, 14, 15, 16, and 17). We found no evidence for private CNVs in any of these centromere-proximal regions (Figure 1A). Based on the results obtained in ZALENDE/EiJ, it would appear that the emergence of Rb races is not associated with large-scale changes in sequence content, at least in the regions of the genome included in the reference assembly.

Novel alleles presented here have already been used to increase the utility of SNP arrays for genotyping of wild mice (Morgan *et al.* 2016a). We provide tools to browse the variants, the underlying read alignments, and the raw reads (see *Data Availability* in *Materials and Methods*). In particular, we provide access to the BWT of the sequencing reads generated from each of these strains. A BWT is a compact and lossless data structure that allows rapid interrogation of the reads in the absence of alignment.

Integration of these two novel genome sequences with the growing catalog of known *M. musculus* genetic variation will provide a valuable resource to researchers using mouse models to study a wide variety of biological processes, including karyotype evolution (Chmátal *et al.* 2014), speciation (Payseur and Place 2007), protein evolution (Karn *et al.* 2008; Stempel *et al.* 2016), gene duplications (Laukaitis *et al.* 2008; Morgan *et al.* 2016b), X-chromosome inactivation (Calaway *et al.* 2013), and disease-associated quantitative traits (Cervino *et al.* 2005; Perez *et al.* 2013; Nicod *et al.* 2016).
